# Minimally invasive orthodontic management: a paradigm shift in interceptive care. Part I - concept and palatally displaced canines

**DOI:** 10.1590/2177-6709.30.6.e25spe6

**Published:** 2026-02-09

**Authors:** Daniela GARIB, Ana Cláudia de Castro Ferreira CONTI, Felicia MIRANDA, Camila MASSARO, Susan SASSAKI, Silvio Augusto BELLINI-PEREIRA, Cibele ALBERGARIA

**Affiliations:** 1 Universidade de São Paulo, Faculdade de Odontologia de Bauru, Departamento de Ortodontia (Bauru/SP, Brazil).; 2 Universidade de São Paulo, Hospital de Reabilitação de Anomalias Craniofaciais, Divisão de Odontologia (Bauru/SP, Brasil).; 3 Clínica Privada (Brasília/DF, Brazil).

**Keywords:** Orthodontics, interceptive, Orthodontics, Cuspid, Tooth eruption, ectopic, Ortodontia interceptora, Ortodontia, Dente canino, Erupção ectópica de dente

## Abstract

**Introduction:**

Conservative approaches in childhood may enhance esthetics and function while reducing the likelihood of complex Phase II orthodontic treatments during adolescence.

**Objective:**

This article aims to introduce the principles of Minimally Invasive Orthodontic Management (MIO) during the mixed dentition period, promoting the concept of “as little intervention as possible”, in accordance to The “Orange July” campaign.

**Methods:**

The core principles of MIO include: (1) early diagnosis and risk assessment; (2) evidence-based, timely, and targeted interventions; and (3) prioritization of patient comfort and psychosocial well-being. Part I addresses the application of MIO to the interceptive management of palatally displaced canines (PDC). Early diagnosis, typically between ages 10 and 12, is based on clinical indicators and radiographic assessment. Major risk factors for PDC include female sex, hypodivergent facial growth pattern, dental spacing, and the presence of associated dental anomalies. Interceptive strategies such as the extraction of the deciduous canine, associated with a success rate of approximately 70%, and adjunctive treatments like rapid maxillary expansion or cervical headgear in Class II patients have demonstrated positive outcomes.

**Results:**

These minimally invasive protocols contribute to simplify Phase II treatment, reducing the risk of maxillary incisor root resorption and improving cost-effectiveness.

**Conclusion:**

The “Orange July” campaign reinforces the importance of early orthodontic evaluation and supports the MIO model as a biologically sound, patient-centered approach to managing developing malocclusions during the mixed dentition phase.

## RESUMO

**Introdução:**

Abordagens conservadoras na infância podem melhorar a estética e a função, reduzindo a probabilidade de tratamentos ortodônticos complexos durante a adolescência, na fase de Ortodontia Corretiva.

**Objetivo:**

Este artigo objetiva introduzir o princípio da Ortodontia Minimamente Invasiva (OMI) durante o período da dentição mista, promovendo o conceito de “mínima intervenção possível”, em linha com a campanha “Julho Laranja”.

**Métodos:**

Os princípios centrais da OMI incluem: (1) diagnóstico precoce e avaliação de risco; (2) intervenções oportunas, pontuais e baseadas em evidência; e (3) priorização do conforto e do bem-estar psicossocial do paciente. A Parte 1 desta série de artigos aborda a aplicação da OMI no tratamento interceptor de caninos ectópicos por palatino (CEP). O diagnóstico precoce, normalmente entre 10 e 12 anos de idade, é baseado em indicadores clínicos e radiográficos. Os principais fatores de risco para CEP incluem: sexo feminino, padrão de crescimento facial horizontal, presença de diastemas, e a presença de anomalias dentárias associadas. Estratégias interceptoras como a extração do canino decíduo, associadas a uma taxa de sucesso de aproximadamente 70%, e tratamentos adjuntos como a expansão rápida da maxila, ou o aparelho extrabucal cervical em pacientes com Classe II, têm demonstrado resultados positivos. Esses protocolos minimamente invasivos previnem a impacção dos caninos, contribuindo para simplificar a segunda fase do tratamento, reduzem o risco de reabsorção radicular do incisivo superior e melhoram a relação de custo-benefício da intervenção ortodôntica.

**Conclusão:**

A campanha “Julho Laranja” reforça a importância da avaliação ortodôntica precoce e apoia o modelo OMI como uma abordagem biologicamente sólida e centrada no paciente para gerenciar más oclusões nas fases precoces do desenvolvimento oclusal.

## INTRODUCTION

The term “conservative” is not new in Dentistry. The concept of minimally invasive dentistry emerged during the transition from the 20th to the 21st century, emphasizing the preservation of healthy tooth structure, risk assessment, early diagnosis, and conservative treatment approaches. The principle of “as little intervention as possible” was advocated by Peters and McLean from the University of Michigan in 2001.^1^ This concept has found significant application in operative dentistry, particularly in the principle of “prevention of extension.”^2^ However, the principles of minimally invasive dentistry are equally applicable to orthodontics, particularly within the context of interceptive treatment. Minimally Invasive Orthodontic Management (MIO) advocates for the use of simple yet effective procedures during the mixed dentition period to guide normal dental and skeletal development, prevent the progression of more severe malocclusions, and reduce the need for extensive orthodontic or surgical interventions in the permanent dentition. The ultimate goal of minimally invasive orthodontic care is to simplify or even eliminate the need for Phase II orthodontic treatment.

MIO aligns with the perspective that less is more-and often better. Its core principles include: 1. Early diagnosis and risk assessment: By using clinical indicators and conservative imaging techniques, clinicians can identify deviations from normal eruption patterns or growth discrepancies at an early stage. In some cases, diagnosis is dynamic and requires longitudinal follow-up to monitor dental development and facial growth patterns. 2. Evidence-based, timely, and targeted interventions: Interventions should be undertaken only when supported by evidence demonstrating long-term benefits. A cost-effectiveness analysis should be conducted for each individual case within a patient-centered care framework. The timing of these interventions is crucial to minimize invasiveness while maximizing therapeutic efficacy. Conservative approaches should adhere to interdisciplinary collaboration and evidence-based practice principles; and 3. Patient comfort and psychosocial well-being: Minimally invasive orthodontic care prioritizes the child’s comfort and emotional development. 

Conservative interventions during the mixed dentition can enhance esthetics and function earlier, potentially preventing the need for more complex Phase II orthodontic treatments during adolescence. Minimally invasive strategies implemented during the mixed dentition period can simplify orthodontic management in the permanent dentition. As orthodontics advances toward precision and individualized care, the minimally invasive approach serves as a unifying concept that encourages clinicians to balance treatment efficacy with the preservation of biological structures. It promotes thoughtful and timely intervention-favoring less but more effective treatment. Clinicians should recognize the value of a shorter and simpler pathway to achieving the best possible occlusion. 

**Figure 1:**
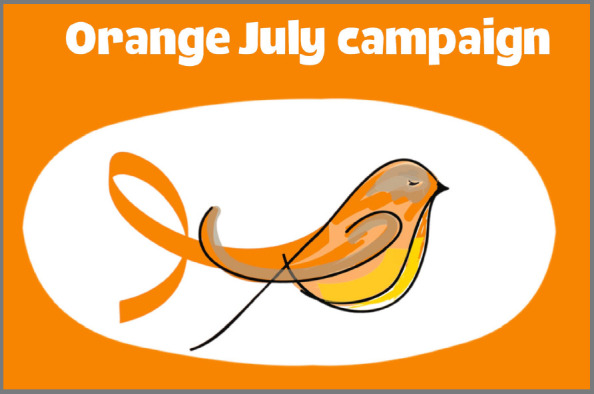
The Orange July campaign advocates for early orthodontic evaluation, emphasizing principles of minimally invasive orthodontic management to support timely, preventive, and patient-centered care.

“Orange July” is a national awareness campaign launched in Brazil to promote early orthodontic assessment, based on the principles of minimally invasive orthodontic management (Fig. 1). The campaign advocates for the first orthodontic evaluation to take place between 5 and 7 years of age, coinciding with the early mixed dentition phase, rather than following the traditional approach of waiting until approximately 12 years of age during the permanent dentition stage. This shift changes the recommendation for the timing of the first orthodontic appointment: on exfoliation of the first deciduous tooth, rather than after exfoliation of the last tooth. By promoting earlier referrals, the Orange July campaign aims to facilitate timely diagnosis, risk assessment, and conservative interceptive interventions, thereby potentially reducing the need for complex and more invasive treatments during adolescence. The campaign targets both general dental professionals and parents/caregivers, emphasizing that early identification of developing malocclusions enables more simple, biologically compatible, and patient-centered care. This initiative reflects a broader trend in orthodontics toward preventive and minimally invasive strategies that emphasize treatment simplification and long-term stability. Prevention represents the highest expression of well-being that individuals can experience in healthcare.

The cost-effectiveness of early treatment includes esthetic and functional improvements, enhanced quality of life, preservation of dental and periodontal tissues, long-term stability, and the simplification of Phase II treatment through reductions in duration, complexity, and invasiveness. Evidence demonstrates a clear cost-effectiveness ratio for the interceptive treatment of three dentofacial irregularities: (1) palatally displaced canines; (2) malocclusions primarily caused by oral habits, including posterior crossbites and dentoalveolar open bites; and (3) Class III malocclusions. This Part I article addresses palatally displaced canines, while malocclusions related to oral habits and the Class III facial pattern are the focus of Parts II and III, respectively.

## PALATALLY DISPLACED CANINES (PDC)

### EARLY DIAGNOSIS

The optimal window for early diagnosis of palatally displaced canines is during the late mixed dentition period, between 10 and 12 years of age.³ In most cases, the canine bud develops in its normal position distal to the maxillary lateral incisors, at the level of the nasal floor.⁴ In this context, the position of the canine during the early and intermediate mixed dentition may mislead even the most attentive clinicians. The disturbance becomes apparent only when the canines begin to erupt. Rather than following a direct path to the dental arch, the ectopic eruption causes the canine to deviate palatally and mesially. In rare cases, the maxillary canine tooth germ is malpositioned even before eruption begins.

**Figure 2:**
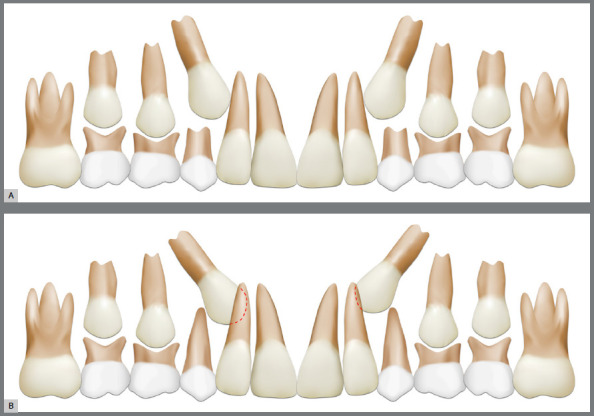
A) Physiologic position of the maxillary canines during the late mixed dentition observed in a panoramic radiograph, illustrating the “ugly duckling” phase; B) Ectopic eruption of the maxillary canines toward the palate, with superimposition of the canine bud image over the lateral incisor root bilaterally.

Early diagnosis begins with clinical evaluation. During the late mixed dentition, clinical indicators of palatally displaced canines (PDC) include asymmetry or severe tipping of the maxillary lateral incisors, as well as the absence of palpable maxillary canine buds on the buccal aspect of the alveolar ridge.^5^ In these cases, a panoramic radiograph should be requested to assess the position of the canine buds relative to the roots of the adjacent lateral incisors. When the contour of the maxillary canine cusp overlaps the root contours of the lateral incisor, the canine is considered ectopic (Fig. 2). Clark periapical radiographs can confirm the palatal position of maxillary canines relative to the roots of the lateral incisors. Unilateral occurrence of palatally displaced canines (PDC) is more common than bilateral involvement.^6^

Two major consequences of palatally displaced canines (PDC) may arise. Ectopic eruption can result in canine impaction, requering complex and invasive procedures in the permanent dentition, such as tooth traction. Additionally, tooth traction can prolong the duration of Phase II orthodontic treatment.^7^ Root resorption of maxillary incisors is another consequence associated with PDC.^8^ In accordance with the principles of minimally invasive orthodontics, interceptive management is recommended upon diagnosis of PDC to simplify comprehensive orthodontic treatment and preserve dental tissues.

### RISK ASSESSMENT

PCD probably has a genetic background. Besides previous citation of familiar history and occurrence in homozygotic twins,^9^ ^,^ ^10^ PDC is more likely to occur in patients with other dental anomalies with a genetic origin.^10^ ^-^ ^12^ To enable preventive management of PDC during the mixed dentition, risk assessments should be conducted early in this developmental phase. Critical risk factors for palatally displaced canines are:

Female gender: Females are two to three times more likely to be affected than males. Consequently, female gender is associated with a higher risk of developing ectopic eruption of maxillary canines compared to males.^11^ ^,^ ^13^

Hypodivergent facial pattern: The majority of PDC cases occur in patients with a hypodivergent facial growth pattern.^6^ Although PDC can also be observed in normodivergent and hyperdivergent patterns, most patients with PDC in our experience are hypodivergent. It is hypothesized that hypodivergent patients possess a greater anteroposterior dimension of the maxilla, providing increased space. Consequently, the maxillary canine can more easily deviate from the labial to the palatal side of the alveolar ridge, encountering less interference from the roots of adjacent teeth.

Positive tooth-arch size discrepancies: Dental arches with spacing exhibit a higher risk for PDC compared to crowded arches.¹⁴ This may be explained by the increased space availability in the dental arch, which facilitates ectopic eruption.

**Figure 3:**
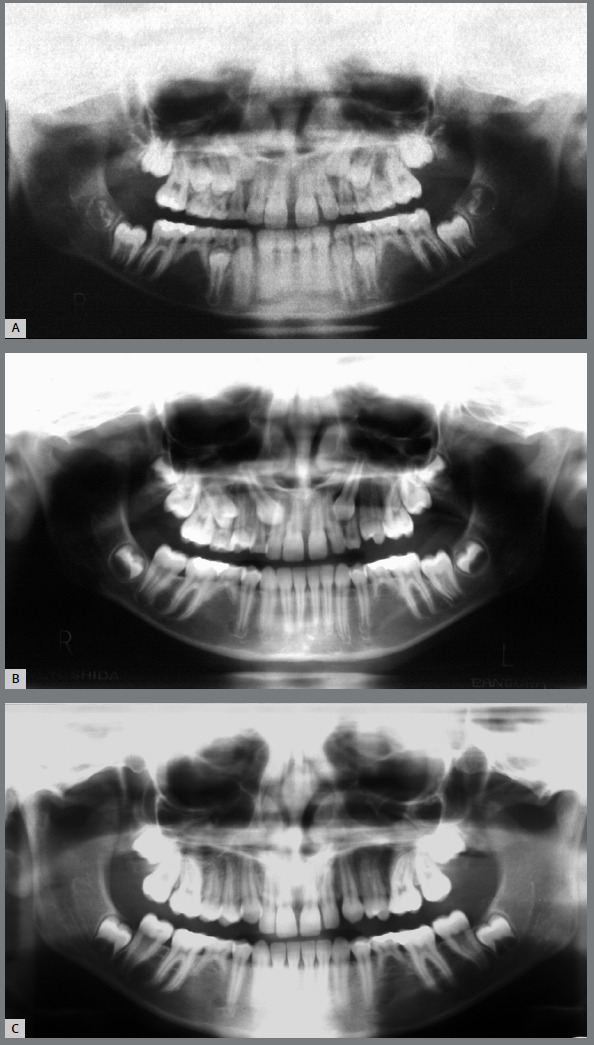
Development of PDC in a patient with agenesis of second premolars. **A)**At the intertransitional period of the mixed dentition, at 9.75 years of age, both maxillary canines were in a normal position. **B)** At the second transitional period of the mixed dentition, at 11.33 years, both maxillary canine were ectopic. The extraction of 53 and 63 was accomplished. **C)**The maxillary permanent canines successfully erupted in the dental arch.

Presence of other dental anomalies: PDC is part of a dental anomaly pattern.^10^ ^-^ ^12^ A shared genetic background may affect multiple teeth with variable expression, resulting in associated dental anomalies. Tooth agenesis, small maxillary lateral incisors, infraocclusion of deciduous molars, and other ectopic tooth eruptions serve as red flags indicating the potential development of PDC in the late mixed dentition.^10^ ^-^ ^12^ When these anomalies are identified during the early mixed dentition, close monitoring of canine eruption between 10 and 12 years of age is essential (Fig. 3).

### MINIMALLY INVASIVE MANAGEMENT STRATEGIES

**Figure 4:**
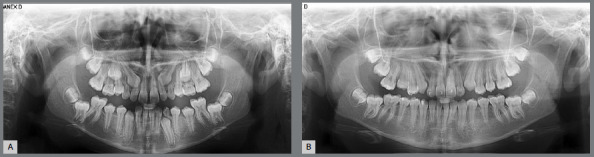
Bilateral case of palatally displaced canine successfully treated with deciduous canine extraction. **A)** At 12 years of age in the second transitional period of the mixed dentition, the superimposition of maxillary canine bud with the root of the lateral incisors revealed the ectopic eruption of maxillary canines. Clark periapical radiographs confirmed the PDC; **B)** One year after the extraction of maxillary deciduous canines at 13 years of age. The maxillary permanent canines successfully erupted, preventing impaction (credit: Dr. Cristiane Nakano).

Minimally invasive management of palatally displaced canines (PDCs) during the mixed dentition period emphasizes interceptive treatment aimed at stimulating proper eruption and preventing the need for more complex surgical or orthodontic interventions later. When PDC is diagnosed during the late mixed dentition, extraction of the predecessor deciduous canine can promote normalization of the canine’s eruption path (Fig. 4).^3^ ^,^ ^15^ The success rate of deciduous canine extraction is approximately 70%.^3^ Prognosis is influenced by patient age and the position of the canine.^16^ Canines that are more mesially displaced have a poorer prognosis compared to those that are only slightly mesially displaced.^16^ In a minimally invasive approach, extraction of deciduous maxillary first molars alongside deciduous canines is not recommended, as the success rate of double extraction is comparable to that of deciduous canine extraction alone.^17^

Following deciduous canine extraction, normalization of the permanent canine eruption should be monitored with a panoramic radiograph taken 10 to 12 months post-extraction.^3^ After deciduous canine extraction, the mean time for permanent canine eruption is approximately 15 months, with a variation of ±6 months.^15^ Unilateral deciduous canine extraction does not cause deviation of the maxillary midline.^18^ Concerns regarding tooth space loss are minimal.^18^ However, a fixed or removable space maintainer with a temporary prosthetic tooth may be used to enhance psychosocial comfort.

Another procedure that influences the eruption of palatally displaced canines during the late mixed dentition is rapid maxillary expansion (RME). RME alone has demonstrated a success rate of 66%, which increases to 80% when combined with deciduous canine extraction.^16^ ^,^ ^19^ In Class II patients with PDC, the use of a cervical headgear appliance has also been shown to increase the success rate of permanent canine eruption.^20^

## CONCLUSIONS

The first orthodontic examination should be conducted no later than the early mixed dentition stage. When interceptive treatment is indicated, the principles of minimally invasive orthodontic care should be applied.

Interceptive management of palatally displaced canines is essential to prevent root resorption of permanent maxillary incisors and to simplify comprehensive orthodontic treatment-thereby reducing patient and family stress, treatment costs, invasiveness, and treatment duration.

## References

[1] Peters MC, McLean ME (2001). Minimally invasive operative care. I. Minimal intervention and concepts for minimally invasive cavity preparations. J Adhes Dent.

[2] McIntyre J (1994). Minimal intervention dentistry. Ann R Australas Coll Dent Surg.

[3] Ericson S, Kurol J (1988). Early treatment of palatally erupting maxillary canines by extraction of the primary canines. Eur J Orthod.

[4] van der Linden FPGM, Duterloo HS (1976). Development of the human dentition.

[5] Ericson S, Kurol J (1986). Radiographic assessment of maxillary canine eruption in children with clinical signs of eruption disturbance. Eur J Orthod.

[6] Sacerdoti R, Baccetti T (2004). Dentoskeletal features associated with unilateral or bilateral palatal displacement of maxillary canines. Am J Orthod Dentofacial Orthop.

[7] Kokich VG, Mathews DP (1993). Surgical and orthodontic management of impacted teeth. Dent Clin North Am.

[8] Ericson S, Kurol PJ (2000). Resorption of incisors after ectopic eruption of maxillary canines a CT study. Angle Orthod.

[9] Leonardi R, Peck S, Caltabiano M, Barbato E (2003). Palatally displaced canine anomaly in monozygotic twins. Angle Orthod.

[10] Peck S, Peck L, Kataja M (1994). The palatally displaced canine as a dental anomaly of genetic origin. Angle Orthod.

[11] Baccetti T (1998). A controlled study of associated dental anomalies. Angle Orthod.

[12] Garib DG, Lancia M, Kato RM, Oliveira TM, Neves LT (2016). Risk of developing palatally displaced canines in patients with early detectable dental anomalies: a retrospective cohort study. J Appl Oral Sci.

[13] Lövgren ML, Dahl O, Uribe P, Ransjö M, Westerlund A (2019). Prevalence of impacted maxillary canines-an epidemiological study in a region with systematically implemented interceptive treatment. Eur J Orthod.

[14] Jacoby H (1983). The etiology of maxillary canine impactions. Am J Orthod.

[15] Naoumova J, Kurol J, Kjellberg H (2015). Extraction of the deciduous canine as an interceptive treatment in children with palatal displaced canines - Part I shall we extract the deciduous canine or not?. Eur J Orthod.

[16] Sigler LM, Baccetti T, McNamara JA (2011). Effect of rapid maxillary expansion and transpalatal arch treatment associated with deciduous canine extraction on the eruption of palatally displaced canines a 2-center prospective study. Am J Orthod Dentofacial Orthop.

[17] Hadler-Olsen S, Sjögren A, Steinnes J, Dubland M, Bolstad NL, Pirttiniemi P (2020). Double vs single primary tooth extraction in interceptive treatment of palatally displaced canines. Angle Orthod.

[18] Bazargani F, Magnuson A, Lennartsson B (2014). Effect of interceptive extraction of deciduous canine on palatally displaced maxillary canine a prospective randomized controlled study. Angle Orthod.

[19] Baccetti T, Mucedero M, Leonardi M, Cozza P (2009). Interceptive treatment of palatal impaction of maxillary canines with rapid maxillary expansion a randomized clinical trial. Am J Orthod Dentofacial Orthop.

[20] Armi P, Cozza P, Baccetti T (2011). Effect of RME and headgear treatment on the eruption of palatally displaced canines a randomized clinical study. Angle Orthod.

